# Ultraviolet radiation and neurodegeneration: molecular mechanisms underlying dual neuroprotective and neurotoxic effects

**DOI:** 10.3389/fncel.2026.1909393

**Published:** 2026-08-13

**Authors:** Shreya Praharaj, Eshika Chakraborty, Gayathri Mahalingam

**Affiliations:** Department of Bio-Medical Sciences, School of Bio Sciences and Technology, Vellore Institute of Technology, Vellore, India

**Keywords:** DNA damage, neurodegeneration, neuroinflammation, oxidative stress, ultraviolet radiation, vitamin D signalling

## Abstract

Ultraviolet radiation exhibits a complex, often contradictory link to neurodegeneration risk/progression, spanning clinically diagnosed diseases [Parkinson’s Disease (PD), Alzheimer’s Disease (AD), Multiple Sclerosis (MS) and Amyotrophic Lateral Sclerosis (ALS)] or intermediate phenotypes (decreased neurogenesis, loss of hippocampal volume). The aim of this review is to highlight the dual impact of UV radiation by collecting and synthesizing experimental (preclinical and translational) evidence as well as epidemiological evidence. Current literature exhibits a clear dichotomy: while Vitamin D is capable of exerting a potent neuroprotective effect via anti-oxidant and anti-inflammatory pathways, chronic and intense UV radiation exposure actually hastens the progression or even drives neurodegeneration via a variety of mechanisms. UV radiation exerts its effects through various interconnected pathways: DNA damage pathways, ROS mediated pathways, vitamin D signaling and the skin-brain axis, which unifies both protective and degenerative effects of UV radiation. Owing to the increasing occurrence of neurodegenerative diseases in the general population, these pathways and mechanisms must be leveraged in future research to develop novel therapeutic and preventive strategies. Investigation of biomarkers linked to certain genetic and environmental factors that could provide a link to predisposition toward neurodegeneration and standardization of UV radiation dosimetry (exposure dose/duration) across experimental or pre-clinical studies must also be prioritized.

## 1 Introduction

Neurodegenerative diseases are characterized as chronic disorders, with hallmark pathology including a gradual loss of neurons and dysfunction of the central nervous system (CNS). In recent years, diseases such as AD, PD, MS and ALS have become increasingly prevalent in the aging population (Muthukumar et al., 2026). Ultraviolet radiation, which is an essential component of solar energy, has been heavily studied for its impact on human health. While certain wavelengths of UV radiation penetrate deep into the dermis and induce collagen degradation causing premature aging, others cause direct DNA damage and mutations leading to cancers such as melanoma (Brar, 2025). Chronic and excessive exposure to UV radiation also could lead to oxidative stress in many eye tissues, leading to serious ocular disorders (Ivanov et al., 2018). Conversely, UV radiation also plays a crucial role in cutaneous production of vitamin D_3_, a known neuroprotectant (MacLaughlin et al., 1982; Holick et al., 1995; Kalueff et al., 2004).

The different neurodegenerative disorders have a range of contrasting pathologies. AD is characterized by cognitive decline, amyloid plaques, neurofibrillary tangles, and decreased neurogenesis in specific regions of the brain (Sengoku, 2020; Grueso and Viejo-Sobera, 2021; Zhou, 2024). With regards to PD, there is a rapid loss of midbrain dopamine neurons along with gradual infiltration of Lewy Bodies into the brain, and also dementia and other age-related plaque pathology (Halliday and McCann, 2010; Absalyamova, 2025). MS, on the other hand, is characterized by focal demyelinated plaques in the CNS, exhibiting heterogeneity due to inflammation, gliosis and neurodegeneration. It is often also preceded by an initial demyelinating event known as Clinically Isolated Syndrome (Popescu et al., 2013; Hart, 2018; Kannan et al., 2025). ALS is a disease characterized by gradual loss of motor neurons in the motor cortex, spinal cord and brain stem, which also exhibits dementia as a pathology (Peters et al., 2015; Tafuri et al., 2015). However, while these different neurodegenerative diseases exhibit distinct etiologies and progress in different regions of the brain, they act upon similar cellular and molecular processes, thus exhibiting certain overlapping pathologies (Teleanu et al., 2022). These disorders share certain common hallmark alterations, such as changes in mitochondrial function, autophagy dysfunction and impaired immune response (Butler et al., 2022). Several studies determined the role of vitamin D_3_ supplementation in targeting these convergent pathways and exerting a potent neuroprotective effect (Zhou et al., 2019; Li, 2025). However, this negative correlation was impacted in certain studies due to confounding factors such as age, adaptation to sunlight, hours spent outdoors, latitude and others (Kravietz, 2017; Davis et al., 2023; Karakis, 2023).

This review analyses the relationship between exposure to UV radiation and neurodegeneration by taking into account both epidemiological and experimental evidence. Epidemiological evidence focused mainly on ambient UV radiation exposure and thus, most evidence was found to provide a negative correlation with neurodegeneration.

Experimental evidence on the other hand focuses on chronic and excessive UV radiation exposure, particularly using animal models as subjects, thus more of a positive correlation is established (Becklund et al., 2010; Kumar, 2011; Romeo, 2013; Han, 2017; Irving et al., 2019; Yoon, 2024). The capability of UV radiation to induce DNA damage and oxidative stress has also been taken into account since that too could lead to neurodegenerative pathologies (Kohen and Nyska, 2002; Heck et al., 2003; Poljšak and Dahmane, 2012; Cadet and Douki, 2018). Experimental evidence regarding vitamin D signaling provides strong mechanistic evidence with respect to neuroprotection and alleviation of neurodegenerative pathologies (Burne et al., 2005; Ito, 2011; Gianforcaro and Hamadeh, 2012; Guo, 2016; Morello, 2018; Bayo-Olugbami, 2022).

Furthermore, this review also elucidates the mechanistic pathways underlying both UV radiation-induced damage and neuroprotection, Emphasis is placed on the skin-brain axis and it’s link to neurodegeneration, either via the vitamin D pathway (Holick et al., 1981, 1995; Bikle, 2011) or the pathogenic HPA-axis activation. While experimental evidence obtained from animal models has been translated to human clinical trials, and certain therapeutic strategies have also been developed for the same, future research must be done in this direction (Becklund et al., 2010; Hart, 2018, 2025; Irving et al., 2019).

## 2 Methodology

To ensure comprehensive and objective evaluation of the dual effects of UV radiation exposure on neurodegeneration, a literature search was conducted using Google Scholar as the primary database to obtain a wide range of peer-reviewed literature, while all the relevant findings were corroborated using structured databases such as PubMed and Scopus. The search encompassed publications ranging from database inception up to May, 2026.

Specific keywords were utilized to collect relevant articles including (“Ultraviolet Radiation” OR “UVB Radiation”) AND (“Neurodegeneration” OR “Alzheimer’s Disease” OR “Parkinson’s Disease” OR “Amyotrophic Lateral Sclerosis” OR “Multiple Sclerosis”). For analysis of mechanistic pathways, additional keywords used were (“Vitamin D Signaling” OR “Reactive Oxygen Species” OR “DNA Damage Pathways” OR “Skin-Brain Axis”).

Articles were selected for inclusion in the review if the publications were in English, contained peer-reviewed original research, epidemiological cohort studies or comprehensive meta-analyses, explicitly studied the neurological outcome of UV radiation exposure or UV radiation-mediated vitamin D synthesis, made use of *in-vivo* murine models, analysed translational evidence or that in relation to human clinical trials.

The literature search was performed ensuring that any evidence relating to Vitamin D supplementation provided a comparative insight or distinction from ambient UV exposure mechanisms. Publications that were non-peer reviewed, focused on dermatological outcome rather than neurodegenerative outcome, or lacking sufficient and validated evidence were excluded from the review. Full-text articles were critically appraised, particularly to analyse the physiological relevance of UV radiation dosing and confounding factors such as adaptation to sunlight, geographical latitude and occupation.

## 3 UV exposure characteristics

Ultraviolet radiation constitutes 5% of terrestrial sunlight and plays a role of great interest in the sphere of photo-immunology. It is an integral portion of the electromagnetic spectrum and is further divided into its own spectrum due to variations in wavelength, majorly involving three regions: UVA, UVB, UVC (Diffey, 2002).

UVA radiation spans wavelengths ranging from 320 to 400 nm and along with a portion of UVB spectrum, is the only radiation that can reach the surface of the Earth (Jin et al., 2022). Previous studies have confirmed a link between sunbed use and melanoma incidence. UVA radiation is a major component of the emissions of these tanning beds and thus it establishes the association of UVA radiation and melanoma (Autier, 1994; Autier et al., 2011; Sola and Lorente, 2015).

UVB radiation has a wavelength ranging from 280 to 320 nm and only 1%–10% of it reaches the Earth’s surface. Due to its shorter wavelength, it has greater energy and thus, is capable of causing a wide range of damage to organic compounds (Narayanan et al., 2010). This radiation is capable of inducing direct DNA damage, for example, via single strand crosslinking by catalyzing the formation of cyclobutene pyrimidine dimers (CPDs) and pyrimidine (6–4) pyrimidone products (64PPs) (Cadet and Douki, 2018). Moreover, in 2003 it was discovered that UVB radiation was capable of stimulating Reactive Oxygen Species (oxygen derived free radicals) production through an O_2_ - dependent reaction involving catalase (Kohen and Nyska, 2002; Heck et al., 2003). When the antioxidant defense mechanism is overwhelmed by ROS generation, it could lead to oxidative stress which could further lead to DNA damage, causing apoptosis and cell death (Poljšak and Dahmane, 2012). DNA damage response, i.e., DNA repair plays a highly important role in neuronal development and maintenance (Madabhushi et al., 2014). However, exposure to the UVB radiation band also aids in endogenous production of vitamin D_3_ which further isomerizes to vitamin D_3_ (MacLaughlin et al., 1982; Holick et al., 1995). It is well established that vitamin D plays a crucial role in normal brain aging (Kalueff et al., 2004). Thus, in this review, most of the evidence collected focuses on the dual role of UVB radiation.

UVC radiation (100–280 nm), stores the highest amount of energy and is capable of causing extensive damage to human health. However, since it is entirely absorbed within the ozone layer, it is not an important area for research for this particular study (Oman, 2022).

## 4 Neurodegenerative outcomes of interest

The following review focuses on four major neurodegenerative disorders: Alzheimer’s Disease, Parkinson’s Disease, Multiple Sclerosis and Amyotrophic Lateral Sclerosis.

The biochemical changes responsible for neurodegenerative diseases typically start in the brain decades before the full-blown disease can be detected and clinically diagnosed. These are considered intermediate phenotypes and include a range of conditions (Davenport, 2023). The medial temporal lobe is one of the primary structures of the brain that undergoes atrophy and is capable of progressing toward AD (Salokhiddinov, 2024). Previous studies have also found significant correlation between loss of hippocampal volume and the symptoms of cognitive impairment in AD (Zhou, 2024). Structural vulnerability of the basal ganglia is indicative of pathology of PD (Li, 2022). The lower brain stem undergoes pathological changes the earliest in many cases of PD (Kingsbury, 2010; Arribarat et al., 2020). In case of MS, chronic active white matter lesions can be used to predict possible progression to the clinical disease (Elliott et al., 2019). This disease can also be predicted using cerebral cortex and deep gray matter pathologies (Benedict et al., 2009). Mild Cognitive Impairment is an important means to predict neurodegenerative disorders such as AD (Grueso and Viejo-Sobera, 2021). Certain biomarkers for ALS such as serum creatinine, albumin and CRP levels could also serve as early manifestations of the disease (Sun, 2020).

This review also focuses on the effect that UV exposure has on the manifestation of these intermediate phenotypes since detecting and diagnosing these conditions plays an important role in progression of the disease.

## 5 Epidemiological evidence

Epidemiological studies usually take into account ambient UV radiation exposure and its effect on the general population in terms of neurological health. A recent study conducted by Yupeng Li et al. aimed to establish this link by examining serum levels of 25-hydroxyvitamin-D in 514 Chinese female centenarians. Higher serum 25(OH)D levels were independently linked to lower risk of AD (Li, 2025). Moreover, a study carrying out analysis across 204 countries regarding the impact of ambient UV radiation levels on dementia incidence (hallmark pathology of AD) found there to be a significant negative correlation between UV radiation and dementia incidence. The protective effect of UV radiation was found to be particularly pronounced across those regions with limited access to vitamin D supplementation (You, 2025).

Most available epidemiological evidence establishes a negative association between reasonable UV radiation exposure and PD. Kravietz (2017) derived PD and UV-B radiation data from relevant national databases and established an age-dependent quadratic association between them. While adults below 70 years of age benefited from moderate to high UV-B exposure due to lower PD risk, people older than 70 years of age faced higher risk of PD incidence due to intense UV-B exposure (Kravietz, 2017). A meta-analysis conducted by Zhou et al. (2019) compared eight studies and indicated that 25-hydroxyvitamin D insufficiency and deficiency were associated with increased risk of PD, while increased sunlight exposure when hand-in-hand with reduced risk of PD. A study conducted in Southern Israel compared 3,343 individuals with PD to 31,324 healthy individuals using solar radiation monitors to measure sunlight exposure over 5 years before PD diagnosis. The result was contradictory to previous studies, where people exposed to excessive radiation had 23% higher PD risk compared to those with moderate sun exposure, however, results varied based on population groups and their adaptation to sunlight (Karakis, 2023). The Huang et al. UK biobank study, a large cohort study, made use of 375,599 participants and took into account the effect of outdoor light exposure while also considering genetic predisposition, physical activity, vitamin D levels and sleep cycles. While vitamin D levels and sleep had a relatively smaller effect on PD incidence, outdoor light exposure provided a strong protective effect across seasons and is a modifiable factor, allowing individuals with higher genetic risk for PD to approach the low genetic risk region (Huang, 2025).

A narrative review states the effect of UV radiation variability due to seasonal fluctuations on the immune function of a fetus during gestation. It was postulated that while the vitamin D-VDR mechanism aided in suppressing genetic elements that caused MS, particularly intense UV radiation, at high geographic altitudes or due to seasonal variation, can override this mechanism and cause genotoxicity that increases the risk of MS (Davis et al., 2023). Maternal immune cells, such as T-lymphocytes play a central role in fetal immunity and are affected by intensity of UV radiation- high intensity radiation could lead to inflammation in the CNS, which is central to the pathology of MS (Davis et al., 2023; Woo et al., 2024). Other epidemiological evidence seems to be contradictory to the previous study. For instance, another study involved MS cases and age-matched controls in 2018. When ambient UV radiation exposure of these subjects was analysed, it was found that areas with high UVB levels were linked to 45% lower MS risk, while for children (5–15 yrs) it rose to 49%–55% lesser risk (Tremlett et al., 2018). Another study conducted in 2019 for participants in the third and fourth surveys of the US radiologic Technologists (USRT) Cohort Study, determined that the risk of MS increased as average lifetime levels of UV radiation in winter decreased. This metric was consistent across all subjects of age less than 40 years (Gallagher, 2019). In 2021, a study was carried out to analyse global distribution of MS incidences and its association with UV radiation exposure and air pollution. A fully adjusted regression analysis established a negative association between MS rates and UV radiation, where MS incidence was higher in countries with a lower UV radiation index (Kazemi Moghadam, 2021).

In a study conducted in 2018 to analyse age-adjusted mortality rates due to ALS found that this particular factor was higher in states at more northern latitudes when compared to states at more southern latitudes (Larson et al., 2018). While it is a well-known fact that theoretical UV radiation potential is greater in the southern latitudes than in the north, it is a poor standard for comparison due to other factors such as whether participants are indoor or outdoor workers, the summer window and other such parameters (Godar, 2005). Thus, a well-informed conclusion cannot be drawn. Another study conducted in Taiwan in 2013 established a negative association between sun exposure and ALS, indicating that UV radiation exposure has a protective effect against ALS incidence (Tsai and Tzu-Chi Lee, 2013).

All the evidence is tabulated in Table 1.

**TABLE 1:** Contains all the epidemiological evidence cited above divided into four neurodegenerative outcomes of interest: (a) Alzheimer’s Disease (b) Parkinson’s Disease (c) Multiple Sclerosis (d) Amyotrophic Lateral Sclerosis; each subsection includes the study design, source of study (specific cohort or population wide study), key findings and the subsequent reference.

Neurodegenerative disorder	Study design	Source of study	Key findings	Authors (year)
Alzheimer’s Disease	Cohort (*n* = 514)	Chinese female centenarians	Higher serum 25(OH)D levels were independently linked to lower risk of AD.	Li, 2025
	Ecological (204 countries)	Global database search	Lower dementia incidence in correlation with high UV exposure	You, 2025
Parkinson’s Disease	Ecological (*n* = 69,010)	French national drug claims database	Moderate UV: lowered risk (<70 years) Intense UVB: higher risk (<70 years)	Kravietz, 2017
	Meta-analysis (*n* = 8)	Literature review and meta-analysis	Decreased PD risk with increased sunlight exposure Increased risk of PD with vitamin D deficiency	Zhou et al., 2019
	Case-control (*n* = 3,343 cases/31,324 controls)	Southern Israel population	23% higher risk of developing PD due to intense UV exposure compared to moderate exposure	Karakis, 2023
	Cohort study (*n* = 375,599; range 37–73 years)	Participants in UK Biobank	Protective effect of outdoor light, overrides a high genetic risk for developing PD	Huang, 2025
Multiple Sclerosis	Cohort study (*n* = 151/235 age-matched controls)	Nurses’ Health Study	In areas with high levels of UVB exposure, 45% lower risk of MS, in children (5–15 years), risk lowered by 49–55%	Tremlett et al., 2018
	Cohort study (*n* = 39,801)	US Radiologic Techniques cohort study	In subjects below 40 years, risk of MS increased as average UV radiation lifetime reduced in winter	Gallagher, 2019
	Ecological study	MS data: Institute for Health Metrics and Evaluation website UV index values: Tropospheric Emission Monitoring Internet Service Website.	MS incidence was higher in countries with a lower UV index.	Kazemi Moghadam, 2021
Amyotrophic Lateral Sclerosis	Ecological study (national mortality data)	US National Vital Statistics System Data	Higher ALS mortality in northern latitudes vs. southern latitudes	Larson et al., 2018
	Spatial analysis (*n* = 1,434)	Taiwanese townships	Negative association between sun exposure and ALS	Tsai and Tzu-Chi Lee, 2013

## 6 Experimental evidence

There is a range of experimental evidence linking UV radiation exposure to neurodegeneration using animal models as well as human tissue samples, providing different types of results to establish this link.

**FIGURE 1:**
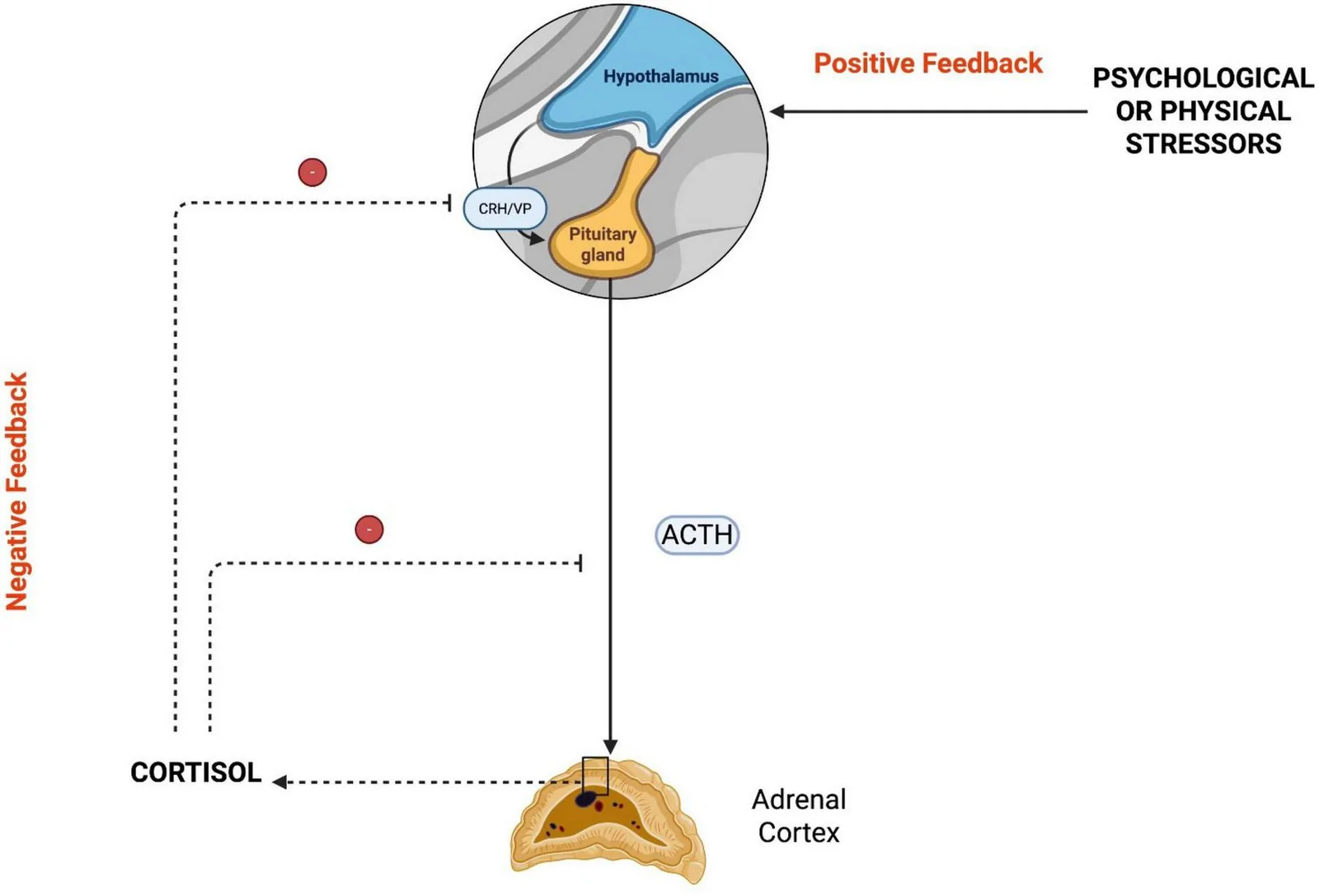
HPA axis involves the hypothalamus, pituitary and adrenal gland. Stress caused by physical or psychological stimuli acts as positive feedback which causes the hypothalamus to release vasopressin (VP) which stimulates the pituitary gland to release the adrenocorticotropic hormone (ACTH), causing the adrenal cortex to release cortisol, which provides negative feedback to the hypothalamus and pituitary to slow hormone production. Created in BioRender. Praharaj (2026) https://BioRender.com/ywa85jt.

The hypothalamus, pituitary gland and adrenal gland undergo direct and indirect interactions via the HPA axis to aid the biological system in handling stress levels (Figure 1).

Constant activation of the HPA axis could lead to hippocampal atrophy, synaptic dysfunction and neuroinflammation, all linked to the pathogenesis of AD (Sharan and Vellapandian, 2024).

**FIGURE 2:**
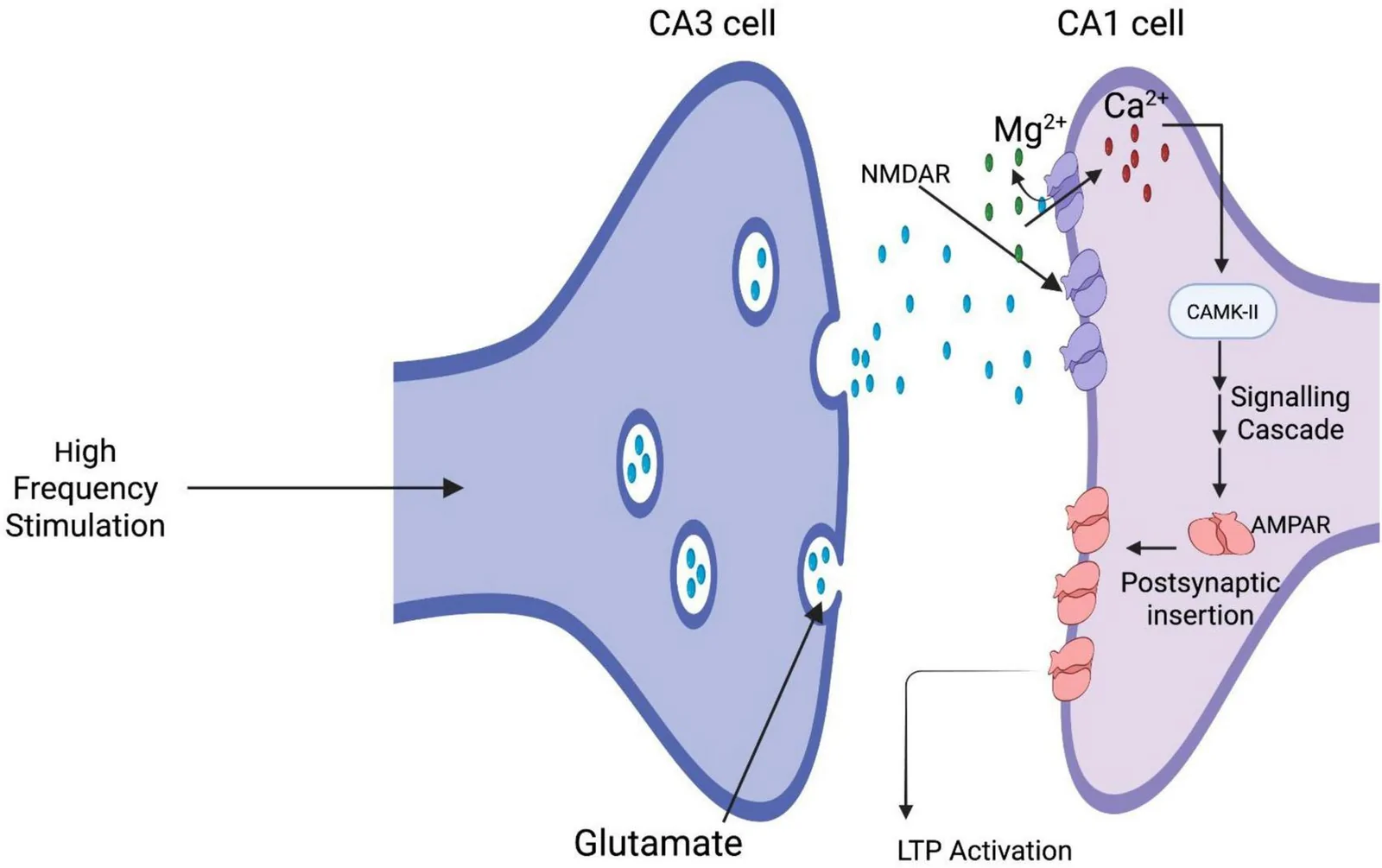
CA3-CA1 synapse is a crucial hippocampal synapse where the CA3 pyramidal cells synapse into CA1 neuronal dendrites. A high frequency stimulation leads to the production of glutamate which in turn activates the N-methyl-D-aspartate (NMDA) receptor, leading to the influx of Ca^2+^ ions and efflux of Mg^2+^ ions. Ca^2+^ ions in turn activate the CAMK-II factor which initiates a signaling cascade that synthesizes the AMPA receptors that undergo postsynaptic insertion and mediate Long-Term Potentiation, which leads to rapid and prolonged activation of the synapses. Created in BioRender. Praharaj (2026) https://BioRender.com/m0qm0ai.

A 2017 study involved study of effects of UV radiation on hippocampal neurogenesis in murine models, particularly tracking DCX^+^ immature neurons in the dentate gyrus. Analysis of these alterations is crucial for quantifying adult neurogenesis and assessing the suppression of new neuron production in a neurodegenerative stage. Immunohistochemistry revealed that 2 weeks of UV radiation exposure of a given group of mice decreased the concentration of DCX^+^ immature neurons when compared to the control group (D’Alessio, 2010; Han, 2017). In another study involving murine models, hippocampal memory was taken into account, particularly Long-Term Potentiation (LTP) at the CA3-CA1 Hippocampal Synapses. LTP plays a highly important role in learning and is also involved in cellular mechanisms governing memory (Figure 2).

The study group mice were irradiated with UV radiation for 6 weeks, after which they underwent a series of behavioral tests. Compared to the control group, UV radiation exposure resulted in a drastic LTP defect and also decreased the number of DCX^+^ immature neurons (Kumar, 2011; Yoon, 2024).

A salient feature of PD is the loss of dopaminergic neurons in the substantia nigra leading to subsequent insufficiency in locomotor function (Absalyamova, 2025). A study conducted in 2013 involved exposing distinct groups of albino rats to varying intensities of light. Toward the end of the irradiation period, the rats were sacrificed and assayed and it was found that there was a significant loss in Tyrosine Hydroxylase (TH) positive neurons along with a subsequent drop in dopamine and its metabolite in the striatum, signifying possible contribution to PD-like degeneration (Romeo, 2013).

A pivotal study carried out in 2019 analysed the effectiveness of UV radiation in suppressing development of experimental autoimmune encephalomyelitis (EAE). EAE is the most commonly used experimental murine model whose pathological features closely resemble that of MS (Constantinescu et al., 2011). It was found that Narrow Band UV-B (UV-NB) light is capable of suppressing EAE in the model using a pathway independent of that of vitamin D, which is a known suppressor of MS (Irving et al., 2019). The landmark paper for this study was provided by Becklund et al. (2010).

When the lens of the eye is exposed to UV radiation between 250 and 400 nm, it could lead to aggregation of crystallin and subsequent clouding of the lens, leading to cataracts (Borges-Rodríguez et al., 2023). UV radiation triggers the production of free radicals known as Reactive Oxygen Species (ROS), which denature the lens proteins causing them to lose their transparency and flexibility over time (Hanafy, 2020). Clinical investigations indicated a link between UV radiation-induced cataracts and AD using murine models. Cataracts were induced in mice via surgery, following which techniques such as immunohistochemistry, immunofluorescence and ELISA were used to detect pathogenesis related to AD. It was found that the cerebral amyloid-beta (Aβ) load in cataract presenting mice was found to be markedly higher when compared to the control group (Geng, 2025).

In earlier studies concerning the pathophysiology of AD, it was found that amyloid plaques (or Aβ plaques) and neurofibrillary tangles were the hallmarks of an aging human brain (Sengoku, 2020). In a study conducted in 2016, on establishing an *in-vitro* model of the blood-brain barrier of mice, it was found that 1,25-dihydroxyvitamin D_3_ aided in enhanced Aβ_1–40_ (major soluble oligomeric form of Aβ) efflux from brain to the blood (Ito, 2011; Guo, 2016). In another study involving animal models, 5XFAD transgenic mice (AD-like mice) were subjected to various different concentrations of vitamin D in their diet (deficient, normal or high) at either early or late stages of the disease. It was found that early supplementation improves both working memory and hippocampal neurogenesis in the animals, while deficient supplementation worsens the hippocampal amyloid load (Morello, 2018).

In the purview of PD, 6-OHDA injected mouse models were used, capable of displaying mild motor impairment just after a week of low dose striatal injection (Slézia, 2023). Vitamin D was provided systemically for several weeks, and it was found that it significantly improved motor performance and also increased the concentration of TH-positive neurons in the substantia nigra and striatum thus improving dopamine production (Bayo-Olugbami, 2022).

There is substantial evidence from animal models regarding the role of Vitamin D as a neuroprotective agent against ALS. An experimental study carried out in 2005 involved the breeding of VDR knockout mice, which then went on to exhibit locomotor and muscular functional impairment without cognitive impairment (Burne et al., 2005). Another one carried out in 2012 proved the usefulness of dietary D_3_ supplementation of G93A transgenic mice (ALS models) to delay muscular decline (Gianforcaro and Hamadeh, 2012). Since the role of UV radiation in endogenous production of Vitamin D has already been established (MacLaughlin et al., 1982; Holick et al., 1995), this is a potential indirect link between UV radiation exposure and neurodegeneration.

The role of ROS in inducing oxidative stress which has an impact on cell death and DNA damage is an accepted phenomenon which could contribute to neurodegeneration (Poljšak and Dahmane, 2012). UV radiation is capable of inducing ROS by directly targeting cellular components or by other photosensitization mechanisms (Kohen and Nyska, 2002; Heck et al., 2003; Jager et al., 2017).

In 2021, an experimental study was conducted without any particular clinical disease in focus. Rat models were exposed to UV radiation for a given period of time after which their brains were analysed for lesions. TUNEL assay was applied, a histological technique used to detect certain types of DNA damage (Mirzayans and Murray, 2020). It was observed that neuronal and cellular degeneration was accompanied by DNA fragmentation. An inverted bell-shaped lesion was created, along with neuronal death whose rate varied according to the layers in which they were located. However, it was also found that expression of Heme Oxygenase (HO)-1 increased immediately in response to UV radiation. This enzyme is responsible for metabolizing heme to combat oxidative stress (Matz, 1996; Nimura et al., 1996; Nakata et al., 2021).

Variant SOD1 coding for superoxide dismutase-1 is the most widely causative locus for ALS (Deng, 1993). Mutant SOD1-G93A is typically induced in transgenic ALS murine models and is translationally relevant (Sau et al., 2007). This mutation is capable of inducing mitochondrial fragmentation and death in spinal cord motor neurons (Song et al., 2013). Oxidative stress is well documented in inducing fragmentation (Liot, 2009).

Amyotrophic lateral sclerosis is a neurodegenerative disease whose main pathology centers around motor neuron pathology and death (Tafuri et al., 2015). Evidence of oxidative damage in ALS patients has been detected in plasma, urine and other samples collected from patients (Weishaupt, 2006; Mitsumoto, 2008). This provides a theoretical link between ROS and neurodegeneration but has not been experimentally validated.

Ultraviolet radiation is capable of causing certain forms of DNA damage, as is elucidated by literature such as (Rastogi et al., 2010; Cadet and Douki, 2018), including DNA strand breaks and oxidative damage.

Phosphorylation of histone variant H2AX leads to the formation of ΥH2AX, an early cellular response to DNA Double Strand Breaks (DSB; Mah et al., 2010). On analysing human-α-synuclein expressing PD mouse models, a high level of ΥH2AX foci was observed, indicating DNA damage (Milanese, 2018).

TDP-43 is a special type of DNA binding protein, found to play an important role in almost all cases of sporadic ALS and frontotemporal lobar degeneration (Arai, 2006; Neumann, 2006). This protein plays an important role in the Non-Homologous End Joining DNA repair pathway, crucial for repairing DSBs. Clearance of this protein in motor neurons leads to increased DSBs (depicted by increased ΥH2AX levels), and impaired NHEJ pathway as is evidenced by human sporadic ALS spinal cords (Mitra, 2019).

The evidence is tabulated in Table 2.

**TABLE 2:** Contains all the experimental evidence cited above divided into four subsections: (a) Direct UV irradiation models (b) Ocular UV models (c) Vitamin D manipulation models (d) DNA damage models (with ROS); each subsection includes the neurodegenerative disease under investigation (AD, PD, MS or ALS), study subject (mouse, rat or human samples), key findings and the subsequent reference.

Experimental models	Neurodegenerative outcome	Study subject	Key findings	References
Direct UV irradiation models	Alzheimer’s Disease	Female C57BL/6 mice	Decreased the concentration of DCX^+^ immature neurons when compared to the control group.	Han, 2017
	Alzheimer’s Disease	Female SKH-1 hairless mice	UV irradiation resulted in a drastic LTP defect and also decreased the number of DCX^+^ immature neurons.	Yoon, 2024
	No Clinical Disease in Focus	Rat models for UV irradiation on cortical surface	Inverted bell-shaped lesions in the brain along with neuronal death	Nakata et al., 2021
Multiple Sclerosis	EAE mouse models for MS	Narrow Band UV-B (UV-NB) light is capable of suppressing EAE in the model (MS murine model) using a pathway independent of that of vitamin D	Becklund et al., 2010; Irving et al., 2019
Ocular UV models	Alzheimer’s Disease	Cataract presenting mouse models	Cerebral amyloid-beta (Aβ) load was found to be markedly higher when compared to the control group.	Geng, 2025
Vitamin D manipulation models	Alzheimer’s Disease	*In-vitro* model of blood-brain barrier of mice	1,25-dihydroxyvitamin D_3_ aided in enhanced Aβ_1–40_ efflux from brain to the blood.	Guo, 2016
	Alzheimer’s Disease	5XFAD transgenic AD mouse models	Early supplementation of Vitamin D improves working memory and hippocampal neurogenesis in the animals.	Morello, 2018
	Parkinson’s Disease	6-OHDA injected mouse models	Systemic vitamin D supplementation improved motor performance, increased the concentration of TH-positive neurons in the substantia nigra and striatum	Bayo-Olugbami, 2022
	Amyotrophic Lateral Sclerosis	VDR knockout mouse models	Locomotor and muscular functional impairment without cognitive impairment.	Burne et al., 2005
	Amyotrophic lateral sclerosis	G93A transgenic mouse models for ALS	Dietary D_3_ supplementation aided in delaying muscular decline.	Gianforcaro and Hamadeh, 2012
DNA damage models (with ROS)	Amyotrophic Lateral Sclerosis	Mutated SOD1-G93A transgenic mouse models	This mutation is capable of inducing mitochondrial fragmentation and death in spinal cord motor neurons.	Song et al., 2013
	Parkinson’s Disease	Human-α-synuclein expressing PD mouse models	High level of ΥH2AX foci was observed, indicating DSBs	Milanese, 2018
	Amyotrophic Lateral Sclerosis	Human-derived neural stem cells and tissues	Clearance of TDP-43 protein in motor neurons leads to increased DSBs, and impaired NHEJ pathway in human sporadic ALS spinal cords.	Mitra, 2019
Environmental/visible light models	Parkinson’s Disease	Sprague-Dawley albino rats	Significant loss in Tyrosine Hydroxylase (TH) positive neurons.	Romeo, 2013

To standardize the comparisons across the above given experimental models, Table 3 depicts the UV radiation wavelengths, intensities and exposure doses across different studies. This data is provided only for preclinical models (animal or cell models) that have been physically exposed to a UV lamp to analyse the results.

**TABLE 3:** Contains standardization metrics, particularly UV radiation wavelengths, intensities and exposure doses for only the preclinical trials in which models were directly exposed to a UV lamp to carry out the study.

Reference	UV radiation wavelength spectrum (nm)	Intensity of UV radiation	Exposure doses
Han, 2017	290–320 nm (UVB)	Not specified	200–400 mJ/cm^2^ (3 days/week for up to 6 weeks)
Yoon, 2024	290–320 nm (UVB)	Not specified	3240 mJ/cm^2^ (3 days/week for 6 weeks)
Becklund et al., 2010	280–360 nm (65% of output in UVB range)	Not specified (13–16 min exposures)	250–500 mJ/cm^2^ (provided every day for 7 days, then for every 2–3 days)
Nakata et al., 2021	365 nm (UVA)	7.96 mW/mm^2^	1.0–4.0 mWh total energy (2.86 × 10^6^–1.14 × 10^7^ mJ/cm^2^ over 1–4 h)
Irving et al., 2019	300–325 nm (narrowband UVB)	Not specified	830 mJ/cm^2^ (administered daily)

## 7 Clinical and translational evidence

The EAE model study for MS (Becklund et al., 2010; Irving et al., 2019) was translated to a randomized, controlled human clinical trial in 2018. MS typically follows an initial demyelinating event known as clinically isolated syndrome (CIS). It is necessary to distinguish CIS from other neurodegenerative disorders to establish therapeutic interventions that prevent progress to full-blown MS (Hart, 2018; Kannan et al., 2025). Phototherapy was provided via UV-NB radiation to half the participants, and by the end of 12 months, 100% participants in the non-phototherapy group and 70% participants in the phototherapy group had converted to MS (Hart, 2018). Though not completely significant in terms of statistics, it does highlight to a certain degree the effect of UV radiation exposure in preventing progression in participants. Preliminary proteomic analysis of patient serum samples several years later provided conclusive evidence that UV-NB radiation exposure led to broad spectrum anti-inflammatory activity by downregulating pro-inflammatory cytokines (TNF, IL1, IL6) and chemokines (CCL3,CCL4) (Hart, 2025).

An experimental study published in 2016, on the subject of addicted sunbed users focuses on detection of striatal D2/D3 receptors for dopamine, which are known to play an important role in the neural processing of rewards stimuli. In addicted tanners, the chronic exposure to UV radiation led to increased dopamine efflux and subsequent reduced binding to D2/D3 receptors (Aubert, 2016; Osugo, 2025). This could potentially lead to fall in D2 receptor levels, which is considered a biomarker for late-stage PD (Xu, 2023). Additionally, oxidative stress due to increased dopamine metabolism in chronic users could lead to dopamine neuronal loss (Song and Kim, 2016).

Another study previously conducted collected nuclear DNA from frontal, temporal and parietal lobes and cerebellum from 11 control subjects and 9 AD subjects. It was found that higher levels of oxidized purine and pyrimidine bases were obtained from AD subjects when compared to controls, indicating oxidative DNA damage (Gabbita et al., 1998). A study in the same realm indicates that 8-hydroxyguanine (DNA damage product) levels are elevated in PD patients when compared to controls, particularly in the substantia nigra (Alam, 1997).

Special focus was also given to impaired DNA repair pathways. The Base Excision Repair (BER) pathway is a crucial pathway required to combat formation of these oxidative DNA bases via DNA glycosylases, particularly 8-oxoguanine glycosylase (Jacobs and Schär, 2012). In compliance with the study mentioned in Gabbita et al. (1998), it was also found that in AD patients, a marked decrease in 8-oxoguanine glycosylase activity was found in the nuclear region of hippocampal and para-hippocampal gyri, temporal gyri and parietal gyri (Lovell et al., 2000).

Recent evidence published in 2024, established high levels of DSBs in both neuronal and non-neuronal cells in post-mortem temporal cortex tissue of patients with Lewy body pathologies (like PD) as well as their increased levels in presymptomatic human-α-synuclein expressing transgenic mice (Koss, 2024), thereby establishing an indirect link between UV radiation and neuronal loss (Rastogi et al., 2010; Cadet and Douki, 2018).

Table 4 summarizes all of the translational evidence elucidated in section “6 Experimental evidence.”

**TABLE 4:** Summarizes all the clinical and translational evidence cited in this article.

Neurodegenerative outcome	Study subject	Key findings	References
Parkinson’s Disease	Addicted sunbed users	Increased dopamine efflux and subsequent reduced binding to D2/D3 receptors. Oxidative stress due to increased dopamine metabolism in chronic users leading to dopamine neuronal loss.	Aubert, 2016; Song and Kim, 2016; Xu, 2023
Alzheimer’s Disease	Nuclear DNA from frontal, temporal and parietal lobes and cerebellum	Oxidative DNA damage in AD patients compared to controls	Gabbita et al., 1998
Parkinson’s Disease	Control and PD brains	8-hydroxyguanine levels are elevated in PD patients, particularly in the substantia nigra.	Alam, 1997
Alzheimer’s Disease	Four brain regions of AD and control subjects (age-matched)	A decrease in 8-oxoguanine glycosylase activity was found in the nuclear region of hippocampal and para-hippocampal gyri, temporal gyri and parietal gyri, indicating BER pathway dysfunction.	Lovell et al., 2000
Parkinson’s Disease	Patients with Lewy body pathologies; presymptomatic human-α-synuclein expressing transgenic mice	High levels of DSBs in both neuronal and non-neuronal cells in post-mortem temporal cortex tissue.	Koss, 2024
Multiple Sclerosis	Human participants exhibiting Clinically Isolated Syndrome (CIS)	100% participants in the non-phototherapy group and 70% participants in the phototherapy group progressed from CIS to MS	Hart, 2018; Kannan et al., 2025

The neurodegenerative outcome of interest (AD, PD, MS) has been highlighted, along with the subject of study (for instance, post-mortem brains, live human clinical trial subjects), key findings and references.

Beyond this, UV radiation is known to be the primary contributor to melanoma, or skin cancer. This oncological consequence seems to share a disease-specific association with neurodegeneration, which can be established with the help of epidemiological evidence. Emerging evidence establishes a strong, reciprocal link between PD and melanoma, where mutations in genes PARK1/4 influence α-synuclein aggregation in both PD and melanoma (Wu et al., 2025). In the realm of ALS a significant increase in mortality was observed among survivors of melanoma (Freedman et al., 2013). However, a surprising association was found in the sphere of AD, where patients with malignant melanoma exhibited a reduced risk of subsequent AD (Ibler et al., 2018). This apparent dichotomy suggests that there might be a genetic or metabolic predisposition toward either uncontrolled cellular proliferation and survival, or cellular damage and apoptosis leading to neurodegeneration.

## 8 Mechanisms linking UVB to neurodegeneration

### 8.1 ROS and DNA damage pathways

UVB radiation is a major factor behind the production of ROS, causing photooxidative damage. The overall mechanism of action involves absorption of photons by endogenous photosensitizers which react with oxygen and produce a range of free radicals capable of damaging a range of cells (Heck et al., 2003; Ivanov et al., 2018).

A majority of the pathways that link DNA damage to neurodegeneration involve oxidative genotoxic stress mechanisms. Neuronal tissue is normally resistant to ROS, and maintains balance between oxidative lesions and subsequent DNA repair pathways. When this balance is disrupted, it leads to the pathology of neurodegenerative diseases (Uddin, 2025).

The major form of oxidative damage involves DNA base modifications such as 8-oxoguanine formation, DNA DSBs or Single Strand Breaks (Rastogi et al., 2010; Allgayer et al., 2013; Cadet and Douki, 2018). Erroneous pairing of this altered base with Adenine leads to excision of this base pair through the BER pathway, that further leads to abasic sites. ROS is linked not only to such oxidative lesions but also DSBs and SSBs (Hegde et al., 2011).

ΥH2AX, as explained above, is a major biomarker that indicates occurrence of DSBs. Once a DSB occurs, it further activates protein kinases such as ATM, which further phosphorylate histone H2AX forming ΥH2AX foci, and a cascade reaction leads to induction of DSB repair pathways such as NHEJ pathway (Georgoulis et al., 2017). Thus, high levels of ΥH2AX, as is observed in Milanese (2018), indicates high levels of DSBs in patients with neurodegenerative diseases.

Mitochondrial damage also plays an important role in the pathogenesis of several neurodegenerative diseases such as ALS (Liot, 2009; Song et al., 2013). Mitochondria is the only cellular organelle in the human cell that possesses its own DNA, and is thus particularly susceptible to attack by ROS. Sustained mitochondrial DNA damage leads to mitochondrial dysfunction, which has been proven to affect glial cells in the central nervous system (CNS; Hollensworth, 2000).

Evidence also suggests that a major mechanism of progressive neurodegeneration involves impaired DNA Damage Response, particularly the BER pathway and NHEJ pathway (major pathway for repair of DSBs in mature neurons). Targeted deletion of polβ, which is a polymerase that aids in DNA repair synthesis in BER, leads to lethality in neonatal neurons (Sugo, 2000). Similarly, deletion of XRCC2 and Lig4, essential for DSB repair in NHEJ pathway, is associated with death of mature neurons (Orii et al., 2006).

**FIGURE 3:**
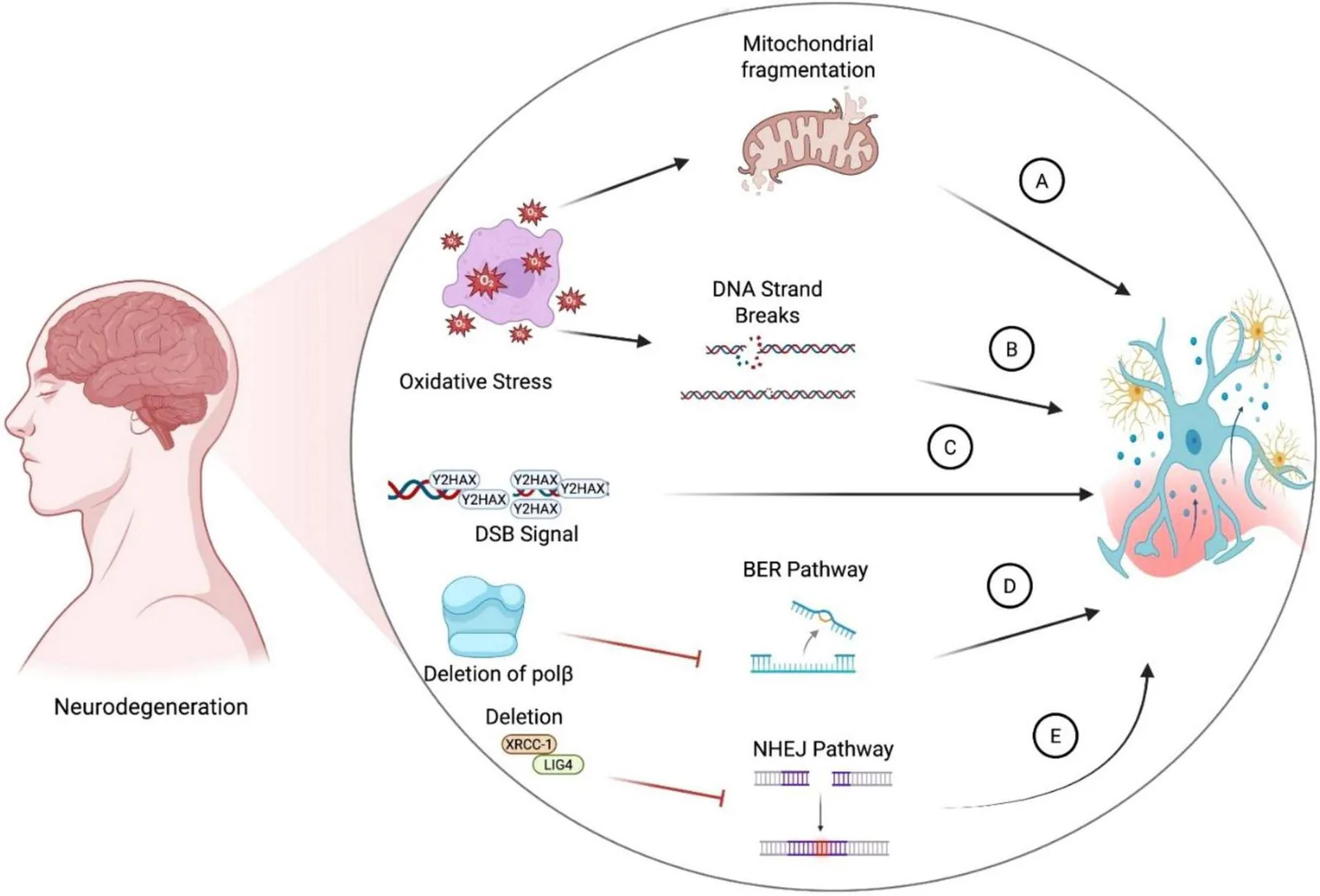
Mechanism of Neurodegeneration due to DNA Damage occurs due to three main pathways: Oxidative Stress caused by ROS could lead to panel **(A)** Mitochondrial fragmentation or **(B)** Single or Double DNA Strand Breaks (DSBs or SSBs); **(C)** Factor ΥH2AX which is a signal for DSBs could increase in concentration indicating neuroinflammation; **(D)** Deletion of polymerase β could inhibiting the Base Excision Repair pathway meant for repairing DSBs leading to neuroinflammation; **(E)** Deletion of XRCC-1 and Lig-4 leads to inhibition of Non-Homologous End Joining pathway which affects DNA repair. Created in BioRender. Praharaj (2026) https://BioRender.com/jzdk9ot.

A flowchart depicting all the above mechanistic evidence is provided in Figure 3.

### 8.2 Vitamin D signaling

When human skin is exposed to UVB radiation, 10%–15% of 7-dehydrocholesterol present in the skin is converted to pre-vitamin D_3_, which further isomerizes to vitamin D_3_ (Holick et al., 1981, 1995). Keratinocytes within the epidermis metabolizes this to active component 1,25-dihydroxyvitamin D_3_ which acts through the vitamin D receptor (VDR; Bikle, 2011).

Vitamin D plays an invaluable role in neuroprotection, with a variety of mechanisms of action that affect both neuronal and non-neuronal cells (Kalueff et al., 2004; Lang et al., 2019). The two main mechanisms include:

#### 8.2.1 Anti-inflammatory activity

Neuroinflammation is the response of CNS to disturbed homeostasis. The CNS is composed of a myriad of reactive components including microglial cells, blood brain barrier, cytokines and their respective signaling pathways, all of which contribute to neuroinflammation (Ransohoff et al., 2015). Vitamin D is capable of suppressing activity of dendritic cells that produce cytokines essential for T-cell differentiation, thus downregulating T-helper cells and subsequent proinflammatory cytokines such as IL-17 and IL21 (Berer, 2000). A study using PD mouse models supplemented with calcitriol (1,25-dihydroxyvitamin D_3_) established that calcitriol not only suppressed pro-inflammatory cytokines but also enhanced anti-inflammatory Treg lymphocytes and suppressed microglial activation (Xie et al., 2024).

#### 8.2.2 Antioxidant activity

Oxidative stress is a major mechanism that leads to neuronal dysfunction and degeneration (Adki et al., 2020). It plays an important role in pathophysiology of neurodegenerative diseases through a variety of mechanisms such as induced oxidation of lipids, nucleic acids and proteins, mitochondrial dysfunction, glial cell activation and amyloid plaque formation (Yaribeygi et al., 2018). Vitamin D displays crucial antioxidant activity by regulating ROS signaling pathways and thus allows normal functioning of the mitochondria, working in conjunction with factors such as Klotho and NRF2, deficiency of this vitamin could lead to signaling instability and dysregulation of ROS signaling causing oxidative stress (Berridge, 2015; Calton et al., 2015). A key mediator for inflammation is inducible nitrogen oxide synthase (iNOS) which is capable of producing nitric oxide (NO). This enzyme is also regulated by proinflammatory cytokines, thus contributing indirectly to neuroinflammation (Sparrow et al., 1994; Cinelli et al., 2020). On studying a rat model of brain inflammation, it was found 1,25-hydroxyvitamin D_3_ is capable of inhibiting iNOS expression, both at mRNA and protein level, thereby also reducing NO levels (Garcion, 1998). Glutathione (GSH) also plays a crucial role in clearing of ROS such as nitric oxide. Analysis of rat primary astrocytes established that 1,25-hydroxyvitamin D_3_ played a crucial role in regulating expression of Υ-glutamyl transpeptidase, which governs GSH metabolism (Garcion et al., 1999).

#### 8.2.3 Genetic alterations in vitamin D receptor (VDR) expression

The neuroprotective effect of vitamin D, as is elucidated in Section 8.2.1 and 8.2.2, depends to a great extent on the expression and proper activity of VDR. Thus, any genetic alterations that could lead to improper expression or silencing of the VDR gene could in turn affect downstream signaling cascades, thereby leading to neurodegenerative pathologies. From a mechanistic perspective, inefficient utilization of Vitamin D due to improper expression of VDR is connected to defective clearance of proteins, mimicking amyloid pathology, while also leading to oxidative stress and calcium dysregulation (Gezen-Ak et al., 2014). This is evidenced by research done by Burne et al. (2005), where VDR knockout mice exhibited loss in locomotor and muscular function even without cognitive decline. Several Single Nucleotide Polymorphisms (SNPs) in VDR have been characterized to play a role in increased neurodegeneration, particularly in relation to AD and PD, such as ApaI, TaqI, BsmI, and FokI (Gatto et al., 2016; Janjusevic et al., 2022). This indicates that even if the systemic vitamin D levels are adequate, improper genetic expression of VDRs, leaves the CNS vulnerable to rapid neurodegeneration.

### 8.3 Skin-brain axis

The skin-brain axis allows for bidirectional communication through which CNS responds to psychological stress leading to a pathological effect for the skin or inversely, how the skin mediates stress to the brain (Paus et al., 2006). However, in the purview of neurodegeneration, this review will only consider the mechanisms by which the skin signals the brain:

#### 8.3.1 Vitamin D signaling

This mechanism has already been discussed in the previous section, where the main pathway of vitamin D_3_ synthesis is triggered due to irradiation of the human skin barrier by UVB radiation (Holick et al., 1981, 1995; Bikle, 2011). This particular pathway confers neuroprotection (refer to the previous section: Vitamin D signaling).

#### 8.3.2 Neuroendocrine (HPA axis) signaling

UVB radiation is capable of upregulating local neuroendocrine axes such as the HPA axis (Slominski et al., 2018). Stress conditions caused by factors such as UV radiation exert their effect mainly through the HPA axis. On receiving a signal induced by stress, the hypothalamus releases corticotrophin releasing hormone (CRH) which travels to the pituitary gland and eventually stimulates the release of proopiome-lanocortin (POMC)-derived neuropeptides such as α- Melanocyte Stimulating Hormone (α-MSH) and adrenocorticotropin hormone (ACTH). ACTH travels to the outer layer of the adrenal cortex and stimulates production of glucocorticoids such as cortisol (Smith and Vale, 2006; Figure 2).

Constant activation that could possibly be brought on by chronic UV radiation exposure could lead to hippocampal degeneration, synaptic dysfunction and neuroinflammation, as has been evidenced by literature particularly pertaining to AD occurrence in mouse models (D’Alessio, 2010; Han, 2017).

## 9 Critical evaluation of literature

### 9.1 Limitations of animal models

While small animal models, particularly murine models, have been key in establishing the mechanistic link between UV radiation exposure and neurodegeneration, there are other limitations that must be recognized, from both a dermatological and neuronal perspective. Firstly, mice possess a thinner epidermis when compared to humans, only 2–3 cells thick, along with a dense layer of fur that must be shaved prior to irradiation (Zomer and Trentin, 2018). Therefore, penetration of UV radiation in mice would differ radically from that of humans. Secondly, mice are nocturnal, thus, while they can accurately act as a model for nocturnal melatonin profiles in humans, they cannot model natural diurnal rhythms (Kennaway, 2019). Hence, they fail to act as the “perfect” human model for neurodegeneration. Moreover, studying the progression of the disease in mouse models is difficult, since most have a lifespan of about 2 years, whereas neurodegeneration takes decades of aging and exposure to specific environmental factors to develop. Several preclinical models mentioned as evidence were developed by overexpressing specific genes to induce neurodegenerative pathologies, which does not accurately mimic human sporadic diseases. All of these factors together deeply affect translatability to human trials and studies.

### 9.2 Inconclusive or contradicting findings

While preclinical models and epidemiological studies provided substantial evidence of the neuroprotective effect of Vitamin D, translating these findings into therapeutic alternatives in human clinical trials has not proven to be successful. Upon vitamin D supplementation, patients with AD showed no significant improvement when placed in randomized controlled trials (RCTs; Chakkera et al., 2022). Meta-analyses confirms mixed findings, mostly indicating small or non-significant effects of supplementation (Choudhary et al., 2026). This suggests that UV radiation could exert a neuroprotective effect beyond vitamin D signaling pathways, or else supplementation is being provided at a later stage of progression of neurodegeneration. Moreover, there is a lack of standardization of the exact dosimetry of UV radiation that causes it to progress from an ambient, neuroprotective agent to a damaging factor. This is because different people exhibit varying responses to ambient radiation, relying on confounding factors such as latitude, altitude, weather, and also biological factors such as melanin and genetic variations (Neville et al., 2021).

## 10 Conclusion

Summarizing the available evidence and mechanisms, it is clear that ambient UV radiation exposure is crucial to ensure normal maintenance of neurological function via factors such as vitamin D, taking into account other confounding factors mentioned in the review. UV radiation exposure gives rise to almost all the functional vitamin D in the biological system, providing neuroprotection via antioxidant and anti-inflammatory mechanisms. The vitamin D signaling pathway comes under an important mechanism linking UV radiation to neurodegeneration called the Skin-Brain Axis (also including neuroendocrine signaling). However, chronic and excessive exposure to UV radiation has been linked via experimental evidence to different neurodegenerative pathologies for clinical diseases such as AD, PD, MS, ALS, via mechanisms of action such as oxidative stress mediated by ROS and DNA damage.

Thus, it is highly important to translate these findings to the general population to control the occurrence of neurodegenerative diseases owing to improper exposure to UV radiation. While experimental evidence has been translated into human clinical trials along with therapeutic strategies, there is potential for further research to surpass the translational gap. There is a need for standardization of UV radiation exposure measurements across preclinical models to enable accurate comparisons. Longitudinal human studies could aid in gaining an understanding regarding cumulative effect of UV radiation exposure in one’s lifetime, along with identification of biomarkers that could point to a predisposition toward UV radiation-induced neurodegeneration for early diagnosis and treatment. Most importantly, the translational capacity of vitamin D supplementation in treatment of neurodegenerative disorders in human population must be studied at a deeper level to establish it’s efficacy. Ultimately, developing preventive and therapeutic strategies would involve systematic analysis of genetic and environmental factors that target individual susceptibility.
